# Blunt traumatic pericardial rupture and cardiac herniation with a penetrating twist: two case reports

**DOI:** 10.1186/1757-7241-17-64

**Published:** 2009-12-15

**Authors:** Peter B Sherren, Robert Galloway, Marie Healy

**Affiliations:** 1 Department of Anaesthesia and Intensive care, The Royal London Hospital, Whitechapel, E1 1BB, UK

## Abstract

**Background:**

Blunt Traumatic Pericardial Rupture (BTPR) with resulting cardiac herniation following chest trauma is an unusual and often fatal condition. Although there has been a multitude of case reports of this condition in past literature, the recurring theme is that of a missed injury. Its occurrence in severe blunt trauma is in the order of 0.4%. It is an injury that frequently results in pre/early hospital death and diagnosis at autopsy, probably owing to a combination of diagnostic difficulties, lack of familiarity and associated polytrauma. Of the patients who survive to hospital attendance, the mortality rate is in the order of 57-64%.

**Methods:**

We present two survivors of BTPR and cardiac herniation, one with a delayed penetrating cardiac injury secondary to rib fractures. With these two cases and literature review, we hope to provide a greater awareness of this injury

**Conclusion:**

BTPR and cardiac herniation is a complex and often fatal injury that usually presents under the umbrella of polytrauma. Clinicians must maintain a high index of suspicion for BTPR but, even then, the diagnosis is fraught with difficulty. In blunt chest trauma, patients should be considered high risk for BTPR when presenting with:

Cardiovascular instability with no obvious cause

Prominent or displaced cardiac silhouette and asymmetrical large volume pneumopericardium

Potentially, with increasing awareness of the injury and improved use and availability of imaging modalities, the survival rates will improve and cardiac *H*erniation could even be considered the 5^th ^*H *of reversible causes of blunt traumatic PEA arrest.

## Background

Cardiac herniation is a significant and potentially fatal complication of BTPR. This is by no means a new problem [1,2] and its occurrence in severe blunt trauma is in the order of 0.4% [3,4]. Despite literature experience dating back to 1864 [5], it is an injury that frequently results in pre/early hospital death and diagnosis at autopsy, probably owing to a combination of diagnostic difficulties, lack of familiarity and associated polytrauma [3,6]. Of those who make it to hospital, and are later diagnosed with BTPR, the survival rate is 36.4% - 42.9% [7]. The high mortality rate is probably a reflection of not only BTPR and cardiac herniation but also the associated injuries [3].

Here, we present two interesting cases of both left and right pleuropericardial ruptures and cardiac herniation. Despite the delay in initial diagnosis, both patients survived, though with varying degrees of disability secondary to related traumatic injuries. The second patient is one of the few reported cases of cardiac herniation and a delayed penetrating cardiac injury secondary to rib fractures.

The common issue echoed throughout our experience and those of others is that of missed or delayed diagnosis. With these cases and literature review we hope to provide further awareness of this injury and clues which can be sought from the clinical presentation and investigations to aid diagnosis.

## Case 1

A 21-year-old male was admitted to a district general hospital accident and emergency department following a moderate speed motorbike accident with the predominant vector of force through the chest and head. Initially when seen by the local ambulance service he was noted to be GCS 15/15, have a high Alveolar-arterial gradient but was cardiovascularly stable. Of note, he could not move or feel his legs.

Management in the district general accident and emergency department followed standard Advanced Trauma Life Support (ATLS) practices. Chest radiograph showed pulmonary contusions on the left but nothing else of significance. He became increasingly agitated and hypoxic and was intubated prior to transfer for computed tomography (CT) scan.

Head CT scans showed an interventricular haemorrhage. Spinal images showed T8/T9 fracture/dislocation with a normal cervical CT. Initial chest CT scans were reported as showing dextracardia and bilateral pneumothoraces; on the left side, the pneumothorax was reported as a possible tension pneumothorax. The possibility of a pneumopericardium was later attributed to an anterior pneumothorax. Abdominal and pelvis CT scans were essentially normal.

As time progressed, persistent hypotension developed despite bilateral tube thoracostomies, fluid challenges and inotropes. The initial working diagnosis of spinal shock was made and a referral was made for further management and neurosurgical intervention for stabilisation of the T8-9 fracture/dislocation.

**Figure 1:**
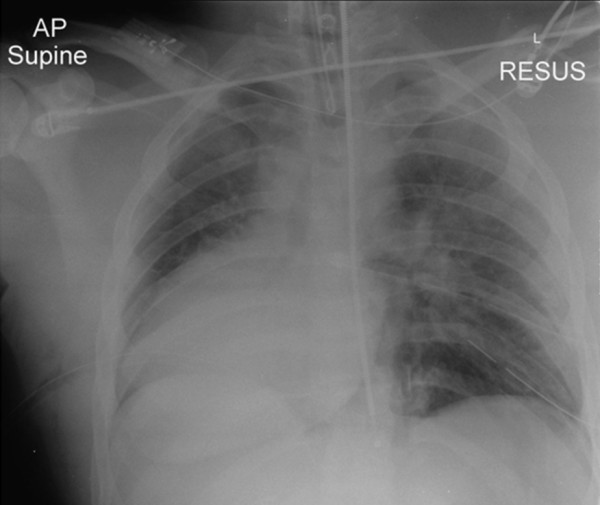
**Plain supine AP chest radiograph showing a prominent, right-sided cardiac silhouette ('boot shaped'); bilateral pulmonary contusions; rib fractures; endotracheal and tube thoracostomies**. With the benefit of hindsight there is the suggestion of a left-sided pneumopericardium surrounded by a faint pericardial contour.

On transfer to our trauma centre, the patient's condition deteriorated; on arrival in our department, he was found to be on a FiO_2 _of 1.0 with PaO_2 _around 10 kPa and requiring high dose norepinephrine and epinephrine to sustain his mean arterial pressure. He was re-trauma called at this stage and plain radiographs were obtained to further ascertain and clarify his injuries (Figure 1).

**Figure 2:**
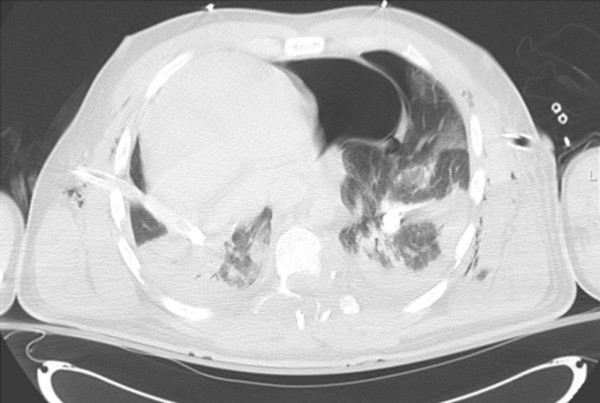
**Axial chest CT demonstrating multiple parenchymal lung contusions; collapsed bilateral haemopneumothoraces; tube thoracostomies; surgical emphysema; large left-sided pneumopericardium; and displacement of the heart into the right hemithorax**.

**Figure 3:**
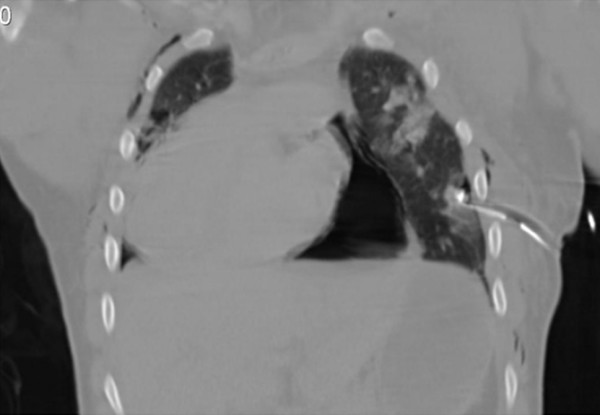
**Coronal chest CT demonstrating most of the axial findings including the prominent pneumopericardium and displacement of the heart into the right hemithorax**.

The finding of dextracardia had been noted previously at the district general hospital and was not thought to be pathological at this stage. A further tube thoracostomy did not improve the hemodynamic status of the patient. The patient was transferred for CT scan where the following images were obtained (Figure 2 and 3).

The CT showed a multitude of head and thoracic injuries. A number of rib fractures and bilateral haemopneumothacaces as well as the aforementioned neuroaxial injuries were all noted. The presence of pericardial air with herniation of the heart into the right hemithorax was also causing concern. At this stage the patient's condition had not improved and, on balance, it was agreed to take the patient to theatre to investigate his thoracic injuries. The patient underwent a clamshell thoracotomy where a 10 cm tear in the right of the pericardium (along the path of the phrenic nerve) was noted with a cardiac herniation through the defect. The heart was noted to be large and dilated. The heart was relocated and the pericardium repaired with interrupted non-absorbable sutures. An intracranial pressure (ICP) bolt was also inserted for monitoring and further management of his traumatic brain injury.

There was an almost immediate reduction in inotrope requirement and the patient was transferred to ICU. His post-op care was complicated by a chest infection and frequent episodes of fast atrial fibrillation secondary to a myocardial contusion, requiring DC cardioversion. He was discharged from ICU after 14 days. Although he was left with a permanent disability from his T8/T9 fracture dislocation, he recovered a good cognitive neurological status and arm strength. With no ongoing cardiovascular problems, he is currently awaiting transfer to a rehabilitation centre.

## Case 2

The second case is that of a 45-year-old male brought into our regional trauma centre by air ambulance. He was the driver in a road traffic collision in which the force vector came through the passenger/left side of the car; unfortunately, the passenger was pronounced life extinct on the scene. On scene, the patient was agitated, moving all limbs and complaining of difficulty in breathing. As a result, he underwent tracheal intubation with drug assistance, bilateral thoracostomies, 750 ml crystalloid and application of a pelvic splint and usual spinal precautions.

On arrival in the department, the initial concern was that of multiple rib fractures, left-sided flail and a large amount of surgical emphysema. Bilateral tube thoracostomies were inserted with 300 mls of blood from the left tube; ventilation/oxygenation improved adequately. Of note, cardiac pulsations were felt when there was a finger sweep of the left pleural cavity.

**Figure 4:**
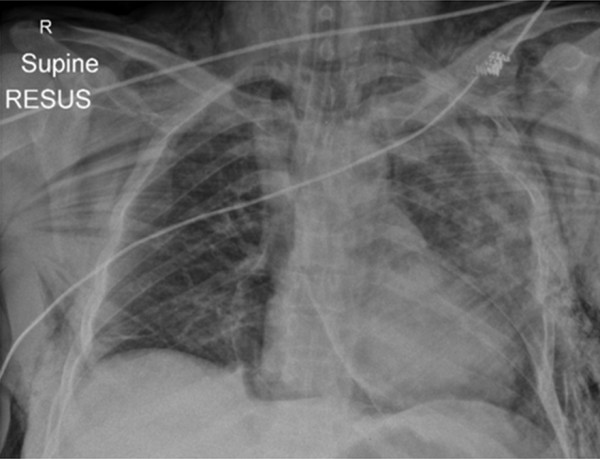
**Plain supine AP chest radiograph showing extensive surgical emphysema; multitude of rib fractures and flail on the left side; bilateral pulmonary contusions and suggestion of a haemothorax on the left side; a rotated 'boot shaped' cardiac silhouette, with clear demarcation of cardiac silhouette from the diaphragm; pneumomediastinum; the pericardial contour is also distinctly visible; endotracheal tube**.

**Figure 5:**
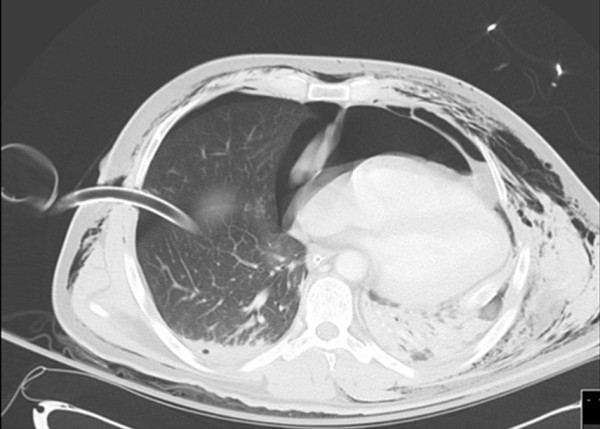
**Axial chest CT showing most of the pathology found on the plain radiograph but also bilateral anterior pneumothoraces; large volume anterior pneumopericardium; tube thoracostomies**.

The chest radiograph showed pneumopericardium, extensive surgical emphysema and improvement in the left-sided haemopneumothorax/right-sided pneumothorax (Figure 4). The evolving problem quickly became that of cardiovascular instability which remained fluid/packed red blood cell responsive throughout. A Focused Assessment with Sonography in Trauma (FAST) scan was negative on two occasions and a pelvic radiograph showed a stable pelvic pubic ramus fracture. Given the negative FAST scans and a period of stability, the patient was taken for a full body CT. The CT chest showed a multitude of injuries, resulting in two further tube thoracostomies being sited (Figure 5). The CT head/spine showed multiple facial and base of skull fractures but no evidence of parencymal or extra-axial bleeds and no fracture or mal-alignment of C/T/L spine. CT abdomen showed a small anterior spleenic laceration with no free fluid in the peritoneal cavity.

Following a joint review of the CT scans and the now-stable patient, no surgical intervention was felt to be needed. The patient had an ICP bolt inserted and was taken to the ICU for further resuscitation and stabilisation.

Sixteen hours post-injury on the ICU, the blood output from the left basal tube thoracostomy started to climb, finally reaching 600 ml/Hr. This was associated with a transfusion requirement, haemodynamic compromise and climbing lactates. At this point, the patient was taken to theatre for a thoracotomy to establish the cause of a likely complex chest bleed.

The following injuries were found and repaired through a left anterior lateral thoracotomy: left-sided longitudinal rupture of the pericardium and cardiac herniation; left ventricular laceration secondary to overlying rib fractures; multiple lung lacerations; and multiple flail ribs. Following insertion of a pericardial and further tube thoracostomies (on suction), the patient was transferred back to ICU for further care.

An eighteen day ICU admission followed, the main issues being that of recurrent atrial flutter and a slow respiratory wean. The respiratory wean was protracted as a result of adult respiratory distress syndrome caused by the primary polytrauma and ventilator-acquired pneumonia.

Over 6 weeks after his initial accident, the patient was discharged home and, all things considered, was doing well in follow up clinic one month later.

## Discussion

### Pathophysiology

Cardiac herniation can occur when there is a significant defect within the pericardial sac. Pericardial tears may involve either the superior/left/right pleuropericardium or the diaphragmatic pericardium. The defect can allow cardiac luxation and, in the case of diaphragmatic pericardial tear, herniation of abdominal contents into the pericardial sac. Clarke et al, one of the largest reviews to date, included a review of 132 cases plus 10 further cases of their own [3]. They found the superior/left/right pleuropericardium were injured in 4%/50%/17% respectively, with the remaining 27% of injuries originating from the diaphragmatic pericardium [3,7]. Of these cases, the rate of cardiac herniation was 28% [3]; however, in a more recent literature search (since 1987), a rate of 64% of the 55 patients with BTPR had cardiac herniation [7]. Defects of the pleuropericardium usually occur vertically along the phrenic nerve; as in our cases [8]. If the tear is large enough, approaching 8-12 cm, the heart can sublux through the defect [9]. The resulting torsion of the great vessels can lead to a form of obstructive cardiogenic shock and cardiovascular instability [8].

### Clinical presentation

As seen with our own experience and those of others there is often a delay in diagnosis of BTPR and cardiac herniation, which is a real concern given that, once recognised, the treatment is simple and effective [10].

The most common mechanism for BTPR are those involved in road traffic collisions and sudden decelerations; particularly those involving a vector of injury from the left side of the chest [3]. The following pattern of associated injuries should also arouse suspicion of BTPR [3]:

• Cardiac - contusions and dysrrthmias (28%). The delayed penetrating cardiac injury as a result of rib fractures, as witnessed in the second case is one of the only reported cases of its kind.

• Chest - multiple rib fractures, haemopneumothoraces and pulmonary contusions almost universally seen.

• Neurological - particularly thoracic spine fractures and spinal cord injuries as well as traumatic brain injuries (32%).

• Abdominal injuries (27%).

• Pelvic and long bone fracture indicative of a high velocity/energy impact (49%).

Given the severity of associated injuries patients usually require invasive ventilation early on. However, if the patient is conscious, they may report symptoms of palpitations, shortness of breath and chest pain as well as angina type pains as a result of coronary obstruction following herniation [2,11].

The main clinical signs, which may be subtle but should be sought, are:

• Signs similar to that of tamponade; in particular that of hypotension, pulsus paradoxus and raised jugular venous pressure (JVP) [2,12]. This may occur early or late depending on the timing of herniation [13]. This haemodynamic compromise may manifest itself despite fluid administration and inotropic support [13].

• Fluctuating haemodynamic parameters, sometimes to the extent of sudden cardiac arrest (often as a result of change in patient's position) should evoke a high index of suspicion of BTPR [14].

• Tachycardia and dysrrthymias may also be seen [11], such as the atrial tachyarrythmias noted in our case.

• Displaced and heaving apex beat [2,8,12].

• A splashing murmur "bruit de Moulin" as a result of the heart moving in a haemopneumopericardium [5,10,12].

### Investigations

Identifying these symptoms and signs in a noisy and stressful trauma environment may well prove difficult. However, there is a multitude of investigations available to most hospitals that can assist in the diagnosis:

• **Electrocardiogram **- may show a tachycardia as well as dysrrthymias particularly those of atrial origin. Also present could be an electrical axis deviation associated with the cardiac herniation and rotation [2,8,12] and a right bundle branch block [9,10]. Ischaemic changes may be noted as a result of coronary artery occlusion by the pericardial band [8,12]. In fact, Rippey et al [12] reported an elevated Troponin I of 9.20 μg/L in a patient later diagnosed with BTPR and cardiac herniation. This was thought to be multifactorial but predominantly as a result of a contusion and coronary insufficiency.

• **Chest radiograph **- as a readily available imaging modality, it is a useful screening tool for BTPR and cardiac herniation. Given the very real chance of the chest x-ray being completely normal, serial films may also be of use to identify any evolving pathology [13]. Findings may include: cardiac silhouette may be unusually prominent ("boot shaped") and demarcated from the diaphragm; pneumopericardium; pneumomediastinum; bowel gas/loops within pericardial sac; prominent pulmonary artery contour; herniation and rotation of the heart into either hemithorax with a possible pericardial sac contour visible distinct to the cardiac silhouette [2,8,10,13]. Associated injuries include haemopneumothoraces, pulmonary contusions, lower lobe collapse/atelectasis/consolidation, surgical emphysema, rib/clavicle/sternal and thoracic spine fractures [13-15].

• **Transthoracic/oesophageal echocardiography and Focused Assessment with Sonography for Trauma **- TTE/TOE have been used with varying reports of success but the sensitivity for diagnosing even large pericardial defects is thought to be low [7,15]. With the presence of surgical emphysema and pneumopericardium, the echographic windows will be poor and, along with operator variability, cannot be relied upon [15]. The importance of echocardiography lies in its ability to rule out other differential diagnoses (such a cardiac contusion or pericardial effusion and possible tamponade) non-invasively and quickly. This can be particularly useful in the patient who is haemodynamically shocked, with a raised JVP, ± reduced heart sounds, and is unresponsive to fluids and inotropic support.

• **Computed Tomography (CT) **- along with its increasing availability and use in the multiply injured trauma patients, CT is also more sensitive for identifying cardiac axis changes and pericardial discontinuity than plain radiographs [13,15].

▪ Characteristic changes for a pericardial rupture include [7,14,15]:

▪ Focal pericardial dimpling and discontinuity

▪ Pneumopericardium

▪ Interposition of lung between: aorta and pulmonary artery; or heart and diaphragm; or right atrium and right ventricular outflow tract

▪ Characteristic changes for a cardiac herniation include [7,15]:

▪ "Empty pericardial sac" sign, air outlining the empty pleuropericardium as a result of cardiac luxution into the hemithorax.

▪ "Collar" sign is the result of compression of the cardiac contour as a result of constriction by the pericardial band caused by the defect.

▪ Associated signs include dilated inferior vena cava (IVC), reflux of contrast into IVC and deformed ventricular silhouette, as well as, secondary signs of tamponade periportal lymphoedema, pericholecystic fluid and ascites.

▪ **Magnetic Resonance Imaging (MRI) **- In haemodynamically stable patients with a suspected BTPR where other imaging modalities have been suggestive but inconclusive, cardiac MRI has been used to clarify its presence [7].

### Management

Once BTPR and cardiac herniation has been diagnosed, treatment is simple and effective. It has even been suggested that, as it is such a rapidly reversible cause of sudden cardiac arrest, there may be a role for post-arrest emergency thoracotomy for select patient groups with blunt chest trauma and positional cardiovascular instability [14].

Video-assisted thoracoscopy has been suggested by some, for the assessment and management of stable patients where there is a lack of diagnostic clarity [8]. Small pericardial defects where cardiac herniation is unlikely, especially those on the left side can be left alone [3,11]. The treatment of choice for tears of the diaphragmatic pericardium, right pleuropericardium, and moderate/large left pleuropericardium defects, is surgical closure [3,10]. Closure of moderate-sized pericardial defects is best achieved by interrupted non-absorbable sutures and larger ones with a mesh prosthesis [3,10,11].

## Conclusion

BTPR and cardiac herniation is a complex and often fatal injury that usually presents under the umbrella of multisystem trauma. The majority of patients will be non-salvageable; where, despite best efforts, the severity of the initial injury results in death prior to arrival in hospital. In the polytrauma patients with severe blunt chest injuries who survive to hospital arrival, the clinician must maintain a high index of suspicion for BTPR. Even with a high index of suspicion, the diagnosis is still fraught with difficulty. However, patients with blunt chest trauma and any of the following signs are exceptionally high risk for BTPR and the need for an urgent operative intervention should be considered:

• Cardiovascular instability with no obvious cause. This instability may be labile and mimic cardiac tamponade, particularly with changes in patient position. A bedside TTE in this setting is a vital tool for exclusion of differential pathology.

• A prominent, possibly displaced cardiac silhouette and asymmetrical large volume pneumopericardium. These signs may show varying degrees of prominence on the plain chest radiograph, if there is uncertainty and the patient's condition allows, a chest CT should be sought as it has been shown to better delineate the injuries. In situations where the patient has a good haemodynamic status and, despite CT, there remains a diagnostic uncertainty, cardiac MR should be considered.

Personal experience and a review of past literature show that, in the majority of cases, it is still an injury diagnosed at autopsy or thoracotomy. Potentially, with increasing awareness of the injury and improved use and availability of imaging modalities, the survival rates will improve and cardiac *H*erniation could even possibly be considered the 5^th ^'H' of reversible causes of blunt traumatic PEA arrest.

## Competing interests

The authors declare that they have no competing interests.

## Authors' contributions

All authors were present at the conception of the project. PBS and RG prepared the draft and all authors were involved in revising the final manuscript. All authors have read and approved the final manuscript.

## Consent

Written informed consent was obtained from the patient for publication of this case report and accompanying images. A copy of the written consent is available for review by the Editor-in-Chief of this journal.
